# Supplementary material for: Kupffer Cells Sense Free Fatty Acids and Regulate Hepatic Lipid Metabolism in High-Fat Diet and Inflammation
Source: Cells. 2020 Oct 8;9(10):2258. doi: 10.3390/cells9102258 (PMC7600268; doi:10.3390/cells9102258)
Supplement: Supplementary file 1 [file cells-09-02258-s001.pdf]

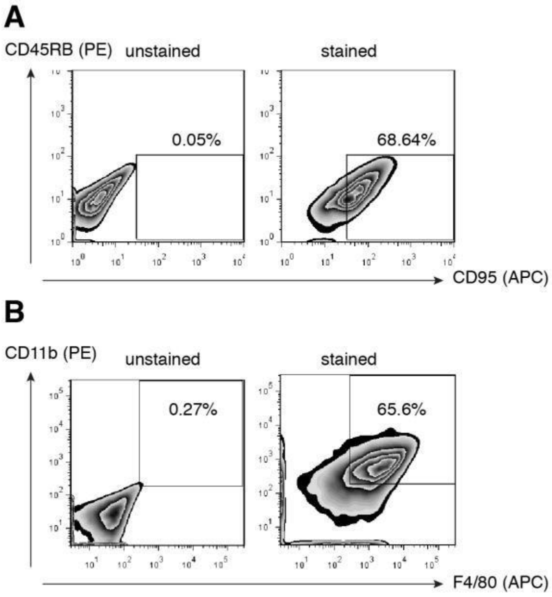

Figure S1. Purity of isolated hepatocytes and Kupffer cells. After isolation, purity of hepatocytes and Kupffer cells was determined by FACS analysis. (**A**) Primary hepatocytes are defined as CD45low/CD95high cells. (**B**) After CD11b MACS isolation, resident Kupffer cells were defined as CD11b/F4/80 positive cells. APC, allophycocyanin; PE, phycoerythrin.

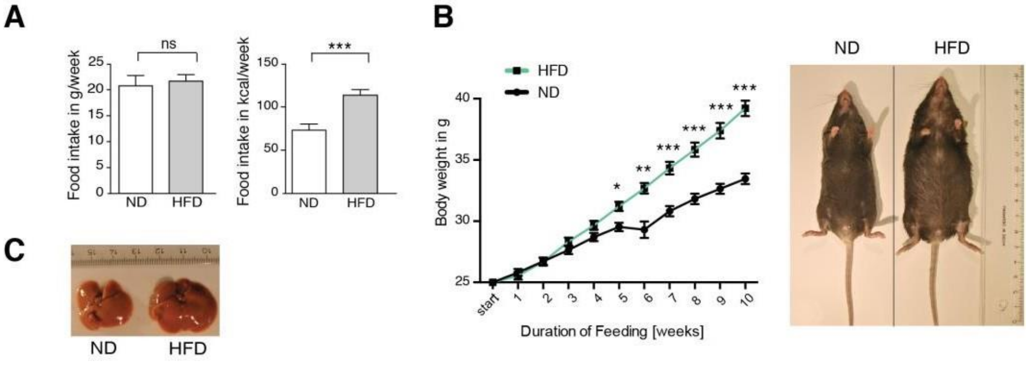

Figure S2. Characterization of ND and HFD mice. (**A**) Comparison of food intake in g/week and kcal/week of ND and HFD mice ($n \geq 4$). (**B**) Averaged body weight during the 10 week feeding period (n = 30/group). (**C**) Photograph of a representative liver from a ND and HFD mouse after 10 weeks of feeding. Data are average ± SEM with at least three independent experiments as indicated. Significance was analyzed with a two-tailed Student's t-test. Statistical significance is indicated as follows: ns, not significant, * $p < 0.05$, ** $p < 0.01$, *** $p < 0.001$.

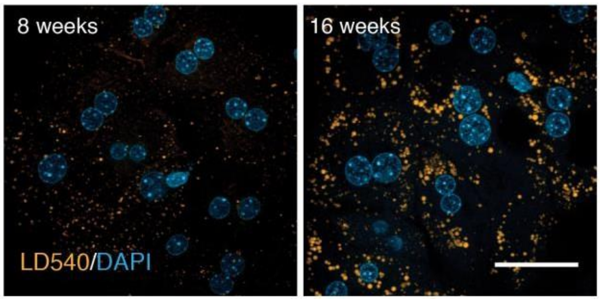

Figure S3. Age-dependent lipid droplet accumulation in hepatocytes. Hepatocytes were isolated from 8 weeks and 18 weeks old mice and stained with LD540 and DAPI 2 h after plating. Scale bar: 50 μm.

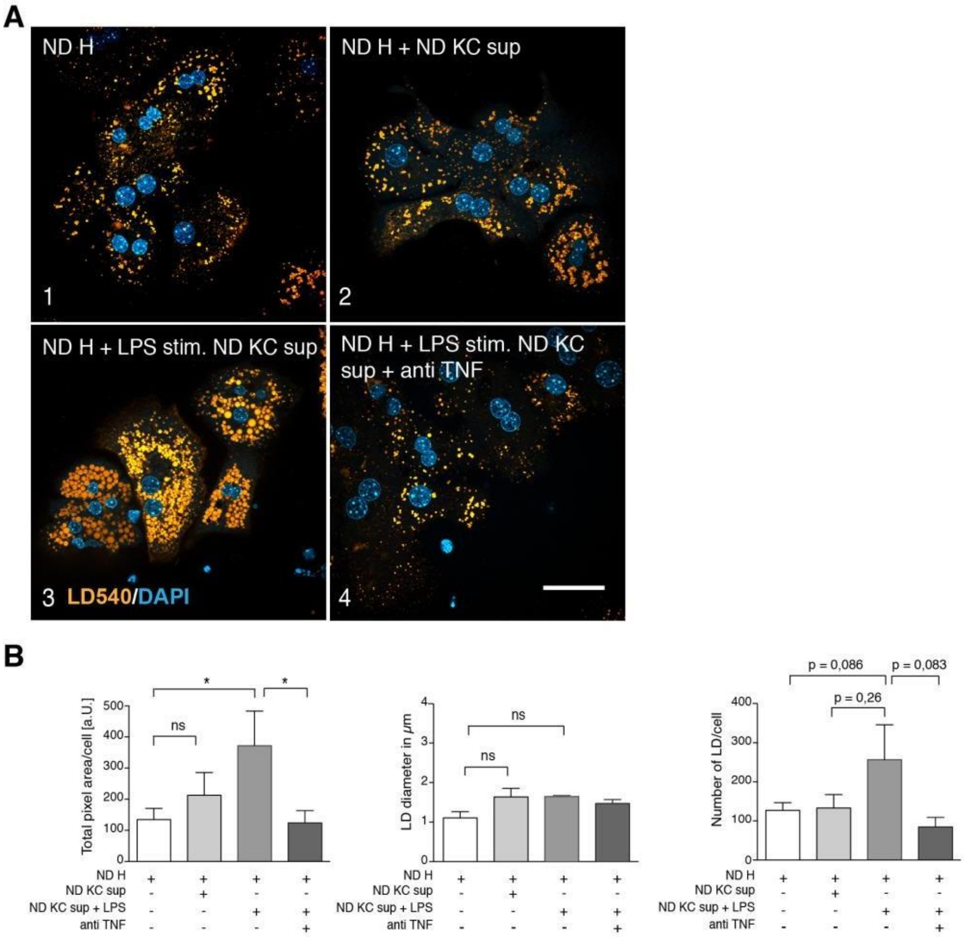

Figure S4. TNF secreted by LPS-stimulated Kupffer cells induces steatotic lipid droplet phenotype in untreated hepatocytes. (**A**) Hepatocytes (H) from normal diet (ND) mice were cultured in the absence (frame 1), or presence of the supernatant of Kupffer cells (KC sup) (frame 2–4), that had either been untreated (frame 2), or treated in vitro with 100 ng/ml LPS (frame 3–4). In frame 4, in addition a neutralizing TNF antibody was added. Cells were stained with LD540 for lipid droplets (LD) and DAPI for nuclei. Scale bar: 50 μm. (**B**) Quantification of total fluorescent LD area per cell and LD size of the various conditions ($n \geq 3$). Data are shown as average with SEM. Significance was analyzed with a two-tailed Student's t-test. Statistical significance is indicated as follows: ns, not significant, * $p < 0.05$.
